# Dopaminergic drug effects during reversal learning depend on anatomical connections between the orbitofrontal cortex and the amygdala

**DOI:** 10.3389/fnins.2013.00142

**Published:** 2013-08-14

**Authors:** Marieke E. van der Schaaf, Marcel P. Zwiers, Martine R. van Schouwenburg, Dirk E. M. Geurts, Arnt F. A. Schellekens, Jan K. Buitelaar, Robbert Jan Verkes, Roshan Cools

**Affiliations:** ^1^Centre for Cognitive Neuroimaging, Donders Institute for Brain, Cognition, and Behaviour, Radboud University NijmegenNijmegen, Netherlands; ^2^Department of Psychiatry, Radboud University Nijmegen Medical CentreNijmegen, Netherlands; ^3^Department of Cognitive Neuroscience, Radboud University Nijmegen Medical CentreNijmegen, Netherlands

**Keywords:** dopamine, striatum, amygdala, OFC, reversal learning, diffusion tensor imaging, bromocriptine, sulpiride

## Abstract

Dopamine in the striatum is known to be important for reversal learning. However, the striatum does not act in isolation and reversal learning is also well-accepted to depend on the orbitofrontal cortex (OFC) and the amygdala. Here we assessed whether dopaminergic drug effects on human striatal BOLD signaling during reversal learning is associated with anatomical connectivity in an orbitofrontal-limbic-striatal network, as measured with diffusion tensor imaging (DTI). By using a fiber-based approach, we demonstrate that dopaminergic drug effects on striatal BOLD signal varied as a function of fractional anisotropy (FA) in a pathway connecting the OFC with the amygdala. Moreover, our experimental design allowed us to establish that these white-matter dependent drug effects were mediated via D2 receptors. Thus, white matter dependent effects of the D2 receptor agonist bromocriptine on striatal BOLD signal were abolished by co-administration with the D2 receptor antagonist sulpiride. These data provide fundamental insight into the mechanism of action of dopaminergic drug effects during reversal learning. In addition, they may have important clinical implications by suggesting that white matter integrity can help predict dopaminergic drug effects on brain function, ultimately contributing to individual tailoring of dopaminergic drug treatment strategies in psychiatry.

## Introduction

Adequate dopamine neurotransmission is well-known to be important for reward and reversal learning and accumulating evidence indicates that the effects of dopamine on such learning implicate the striatum. Consistent with current theoretical modeling work (Badre and Frank, 2012), pharmacological functional magnetic resonance imaging (fMRI) studies have revealed dopaminergic drug effects on striatal BOLD signals during reversal learning (Cools et al., 2001, 2007; Dodds et al., 2008; Van Der Schaaf et al., 2012). In addition, positron emission tomography (PET) studies have demonstrated that reversal learning depends on striatal dopamine synthesis capacity (Cools et al., 2009) and D2 receptor availability in the striatum (Groman et al., 2011).

However, the striatum does not act in isolation and reversal learning is also well-accepted to depend on the interaction between striatum, orbitofrontal cortex (OFC) and amygdala (Iversen and Mishkin, 1970; Jones and Mishkin, 1972; Holland and Gallagher, 2004; Schoenbaum et al., 2009; Murray and Wise, 2010). This interaction is thought to be modulated by dopamine. Specifically, medium spiny neurons in the striatum and amygdala that receive glutamatergic projections from limbic and cortical regions also receive converging dopaminergic projections from the midbrain (Pennartz et al., 1994; Rosenkranz and Grace, 2002a; Sesack et al., 2003; Haber and Knutson, 2010). Animal studies have suggested that the effects of dopamine on these glutamatergic inputs are receptor specific, such that orbitofrontal inputs to the striatum are modulated by D2 receptor stimulation (Del Arco et al., 2007; Grace et al., 2007; Del Arco and Mora, 2009; Sesack and Grace, 2010) while orbitofrontal inputs to the amygdala and amygdala inputs to the striatum are modulated by D1 receptor stimulation (Rosenkranz and Grace, 2002b; Ambroggi et al., 2008; Sesack and Grace, 2010). These observations have led to the suggestion that dopamine regulates the degree to which the striatum, amygdala and OFC interact to integrate information about reward value, motivation and expectation and to ultimately facilitate adaptive and flexible behavior (Grace et al., 2007; Haber and Knutson, 2010; Pennartz et al., 2011).

Here we aim to provide evidence for such network effects of dopamine during human reversal learning by revisiting our recent pharmacological fMRI study that showed dopaminergic drug effects on striatal BOLD signal during reversal learning (Van Der Schaaf et al., 2012). Specifically, we ask whether these previously reported effects of dopamine on the striatum during reversal learning are associated with anatomical connectivity in an orbitofrontal-limbic-striatal network, as measured with diffusion tensor imaging (DTI). A demonstration that drug effects are associated with individual differences in anatomical connectivity will not only address the question about whether dopamine's effects are associated with an orbitofrontal-limbic-striatal network of regions, but will also help elucidate individual trait factors that contribute to the known large variability in dopaminergic drug effects (Cools and D'esposito, 2011). Thus, individual differences in anatomical connections between the OFC, amygdala and striatum might predict the extent and direction of dopaminergic drug effects on reversal learning.

DTI is a non-invasive method to measure structural connectivity in humans. It measures the diffusion of water in tissue, which depends on the tight packing of cellular axons and myelin sheets that encapsulate the axon fibers. Two measures are generally obtained; fractional anisotropy (FA) and mean diffusivity (MD). FA is a measure of the directionality of water diffusion and has been associated with dense coherent bundling and myelination of axons. MD reflects the general presence of barriers to free diffusion and has been associated with overall cell density. Collectively, FA and MD provide information on the microstructural integrity and communicational efficacy of white matter fiber bundles (Beaulieu, 2002; Thomason and Thompson, 2011).

The hypothesis that individual differences in functional effects depend on anatomical connectivity as measured with DTI is grounded in prior work linking anatomical connectivity with individual differences in functional effects (Boorman et al., 2007; Cohen et al., 2008; Harsay et al., 2011; Samanez-Larkin et al., 2012). In addition, we have previously shown that dopaminergic drug effects on striatal BOLD signals during attention-shifting are associated with white matter integrity of dorsal fronto-striatal-thalamic pathways (Van Schouwenburg et al., 2013). These results concur with the known role of dorsal fronto-striatal-thalamic pathways in cognitive functions such as attention shifting (Dias et al., 1996). By contrast, reversal learning depends on a ventral orbitofronto-limbic-striatal network (Dias et al., 1996). In the present study we used a fiber based approach (Mandl et al., 2012) to substantiate the observation that dopaminergic drug effects can be predicted from anatomical connectivity, while also showing the neuroanatomical specificity of such findings. Based on the literature reviewed above, we predict that drug effects on striatal BOLD signal during reversal learning will depend on a ventral orbitofronto-limbic-striatal network and not on a dorsal fronto-thalamic-striatal network.

A final aim of this study was to assess the receptor specificity of the effects (Feldman et al., 1997). As described above, work with experimental animals has suggested that the dopaminergic modulation of interactions between the OFC, amygdala and striatum is dopamine receptor specific (Rosenkranz and Grace, 2002b; Del Arco et al., 2007; Grace et al., 2007; Ambroggi et al., 2008; Del Arco and Mora, 2009; Sesack and Grace, 2010). In addition, it has been demonstrated that reversal learning in monkeys specifically depends on D2 and not D1 receptor functioning (Lee et al., 2007). To address this issue in humans we employed a coadministration design. All subjects were scanned on four occasions: after administration of placebo; after administration of the dopamine D1/D2 receptor agonist bromocriptine; after administration of the dopamine D2 receptor antagonist sulpiride; and after combined administration of both sulpiride and bromocriptine. If drug effects are mediated by D2 receptors, then any significant effect of bromocriptine relative to placebo should be abolished by coadministration of sulpiride. If effects of bromocriptine are mediated by D1 receptors, then they should not be abolished by coadministration of sulpiride.

## Methods

### Subjects

The present study represents an extension of a previously published pharmacological fMRI study (Van Der Schaaf et al., 2012) with diffusion tensor images that were acquired from the same subjects during an intake session prior to the drug sessions. For this study, 28 healthy right handed volunteers with no relevant medical/psychiatric history 3 years prior to testing were tested after a medical screening [for screening procedure and exclusion criteria see Van Der Schaaf et al. (2012)]. They gave written informed consent approved by the local ethics committee (Commissie mensgebonden onderzoek, Arnhem-Nijmegen, number 2008/078, date 09-09-2008) and were compensated for participation. In total, 8 subjects were excluded from the fMRI analysis due to personal issues (1), technical issues (4), excessive head movement (2) and insufficient practice of the Dutch language (1) [see Van Der Schaaf et al. (2012) for further details on these exclusions]. Complete datasets including both DTI and all four fMRI sessions were available for twenty subjects (10 males, mean age: 22.7, range: 18.9–29.1).

### Procedures and pharmacological design

Subjects were tested on four occasions, separated by at least 1 week. They were tested after oral intake of placebo, bromocriptine (Parlodel, Novartis®, 1.25 mg), sulpiride (Dogmatil, sanova-aventis®, 400 mg), and a combination of bromocriptine and sulpiride (sulpiride was administered 30 min prior to bromocriptine). Administration was randomized according to a counterbalanced, placebo controlled, double blind, double dummy design. The reversal learning task started 2¼ h after first drug intake with a duration of 60 min. Blood pressure, heart rate and subjective mood ratings [visual analog scales (Bond and Lader, 1974)] were taken 30 min before, 2 h after and 6 h after first drug intake. Blood samples were taken to determine the change in prolactin levels (Fitzgerald and Dinan, 2008) and were taken 30 min before and 2 h after first drug intake. Background neuropsychological tests (block completion, number cancellation, verbal fluency and digit span) were assessed 5 h after first drug intake. Drug effects on physiological measures were as expected with prolactin increases after intake of sulpiride and combined administration and prolactin and systolic blood pressure decreases after intake of bromocriptine. Analyses of the questionnaires and background neuropsychology are described in our previous report and revealed no significant drug effects on mood or general cognitive functioning. For further details on the screening and session procedures, physiology, mood and background neuropsychology see (Van Der Schaaf et al., 2012).

### Reversal learning task

On each trial, subjects were presented with two simultaneously presented vertically adjacent stimuli, a face and a scene (location randomized). One of these stimuli was associated with reward and the other with punishment. One of the stimuli was selected by the computer (highlighted with a black border) and subjects were asked to predict the outcome associated with this preselected stimulus. After the prediction, indicated with a right index or middle finger button press (counterbalanced across subjects), the actual outcome was presented (100% deterministic). Note that these outcomes did not depend on subjects' responses but were directly coupled to the highlighted stimulus. The stimulus-outcome contingencies reversed after 4, 5, or 6 consecutive correct predictions. Such reversals were signaled by either an unexpected punishment (presented after a previously rewarded stimulus was highlighted) or an unexpected reward (presented after a previously punished stimulus was highlighted). On the trials directly following these unexpected outcomes (reversal trials), the same stimulus was highlighted again such that requirements for motor switching and prediction updating were matched between reward and punishment conditions. Accuracy on these reversal trials reflects how well-subjects updated stimulus-outcome associations after either unexpected rewards or unexpected punishments. The dependent variables used for the current report were striatal BOLD signaling during unexpected outcomes and the proportion of correct responses on reversal trials (see below).

### Image acquisition and preprocessing

Structural images were collected before the start of the experiment during screening using a 3-tesla Siemens MRI scanner with an 8 channel head coil. For each subject, a high resolution T1-weighted MP-RAGE anatomical scan (TE/TR = 3.03/2300 ms, flip angle = 8°, *FOV* = 256 mm × 256 mm × 192 mm, voxel size = 1 mm isotropic, GRAPPA acceleration factor 2) was obtained. Diffusion tensor images were acquired using a twice refocused spin-echo-planar imaging sequence to reduce spatial distortions caused by eddy currents (Reese et al., 2003). Sixteen subjects were scanned with the following protocol: 64 slices interleaved acquisition mode (TE/TR = 89/6700 ms, flip angle = 90°, *FOV* = 220 mm, voxel size = 2.2 mm isotropic). Acquisition consisted of 7 images without diffusion weighting (*b* = 0) and 61 images with diffusion weighting (*b* = 1000 s/mm^2^) applied along the non-colinear directions. Four subjects were scanned with slightly modified protocol in which the TR was 8500 ms and images were acquired with partial instead of full Fourier with a slightly lower band width.

Raw diffusion weighted imaging (DWI) data were pre-processed using in-house software (Zwiers, 2010). The DTI images were realigned using rigid body transformations and mutual information as a cost function (SPM8). Susceptibility induced echo-planar imaging distortions were corrected by warping the images along the phase-encoding direction to the distortion-free T1 reference images (Studholme et al., 2000) using an in-house developed implementation (Visser et al., 2010). Diffusion tensors were then estimated using a robust artifact-insensitive compute algorithm (Zwiers, 2010). FA and MD measures were computed from the diffusion tensor eigenvalues. FA and MD maps were normalized to the T1 ICBM-template (MNI-space) using the unified segmentation parameters of the co-registered structural image. Images were then smoothed with a Gaussian kernel of 8 mm full width half maximum and masked with a full brain mask. Imaging parameters, pre-processing and analysis of the functional images, obtained during the drug sessions, are described elsewhere (Van Der Schaaf et al., 2012).

### General analysis strategy

In our prior work we reported dopaminergic drug effects on striatal BOLD signal during reward and punishment reversal learning (Van Der Schaaf et al., 2012). These BOLD effects were centered on the ventral lateral putamen, a region that receives convergent inputs from both OFC and the amygdala (Draganski et al., 2008; Haber and Knutson, 2010). Here, we revisit our data and ask whether the observed dopaminergic drug effects on striatal BOLD signaling is associated with anatomical connections between the striatum, OFC and amygdala. Thus, we investigated individual differences in white matter integrity of anatomical pathways in an orbitofronto-limbic-striatal network, as indexed by diffusion tensor images that were acquired from the same subjects during an intake session prior to the drug sessions.

We used a fiber-based approach (Mandl et al., 2012) and focused on three anatomical white matter pathways of interest—(1) a pathway connecting the OFC with the striatum (Ongür and Price, 2000; Ogar and Gorno-Tempini, 2007; Haber and Knutson, 2010; Balleine et al., 2011), (2) a pathway connecting the amygdala with the striatum (Robbins et al., 1989; Everitt et al., 1991; Ambroggi et al., 2008), and (3) a pathway connecting the OFC with the amygdala (Baxter et al., 2000; Stalnaker et al., 2007)—, and one anatomical white matter pathway of no interest for control purposes [a pathway connecting the dorsal PFC (dPFC) with the striatum (Haber and Knutson, 2010)]. As described in the introduction, this additional pathway was included to demonstrate specificity of the effects to orbitofronto-limbic-striatal pathways, involved in reward processing and stimulus-outcome valuation. Thus, we anticipated that any effects would not extend to dorsal fronto-striatal pathways that have instead been associated with more cognitive processes and motor control (Alexander et al., 1990; Haber and Knutson, 2010). These study-specific anatomical volumes of interest were first created using probabilistic tractography (see probabilistic tractography section below) and average FA and MD values were extracted from each pathway. These FA and MD values were then used as independent predictor variables in multiple regression analyses with the drug-related change in striatal BOLD signal during reversal learning as the dependent variable (see statistical analysis section below).

### Dependent variable I: selection of striatal bold signal

Striatal BOLD signal was extracted for each drug session from the locus that exhibited the significant drug effect during reversal learning, as reported previously (Van Der Schaaf et al., 2012). This drug effect was centered on the left ventral putamen (*x, y, z* = −22, 18, 4, *p*_fwe_ = 0.03) (Figure 2A) and reflected opposite modulation by the dopamine receptor antagonist sulpiride and the dopamine receptor agonist bromocriptine of BOLD signal change during unexpected relative to expected outcomes. Mean beta estimates from this peak voxel were extracted with MarsBar software (Brett et al., 2002) for each drug session. The use of such a functionally defined timeseries is justified because the aim of our investigation was to account for variability in exactly this signal. Drug-related change in the extracted beta-values (representing signal during unexpected vs. expected outcomes) was then used as a dependent variable in linear regression analysis with the DTI-measurements as predictor variables (see below).

### Dependent variable II: selection of the behavioral values

The behavioral measures of interest were the valence-dependent and valence-independent reversal learning scores. These were calculated by computing, respectively, the difference between, and the average of the proportion of correct responses on reward and punishment reversal trials. The accuracy scores were arcsine transformed [2 × arcsine($\sqrt{\text{x}}$)] as is appropriate when the variance is proportional to the mean (Howell, 1997).

### Probabilistic tractography: selection of frontal-striatal-limbic pathways

**Figure 1:**
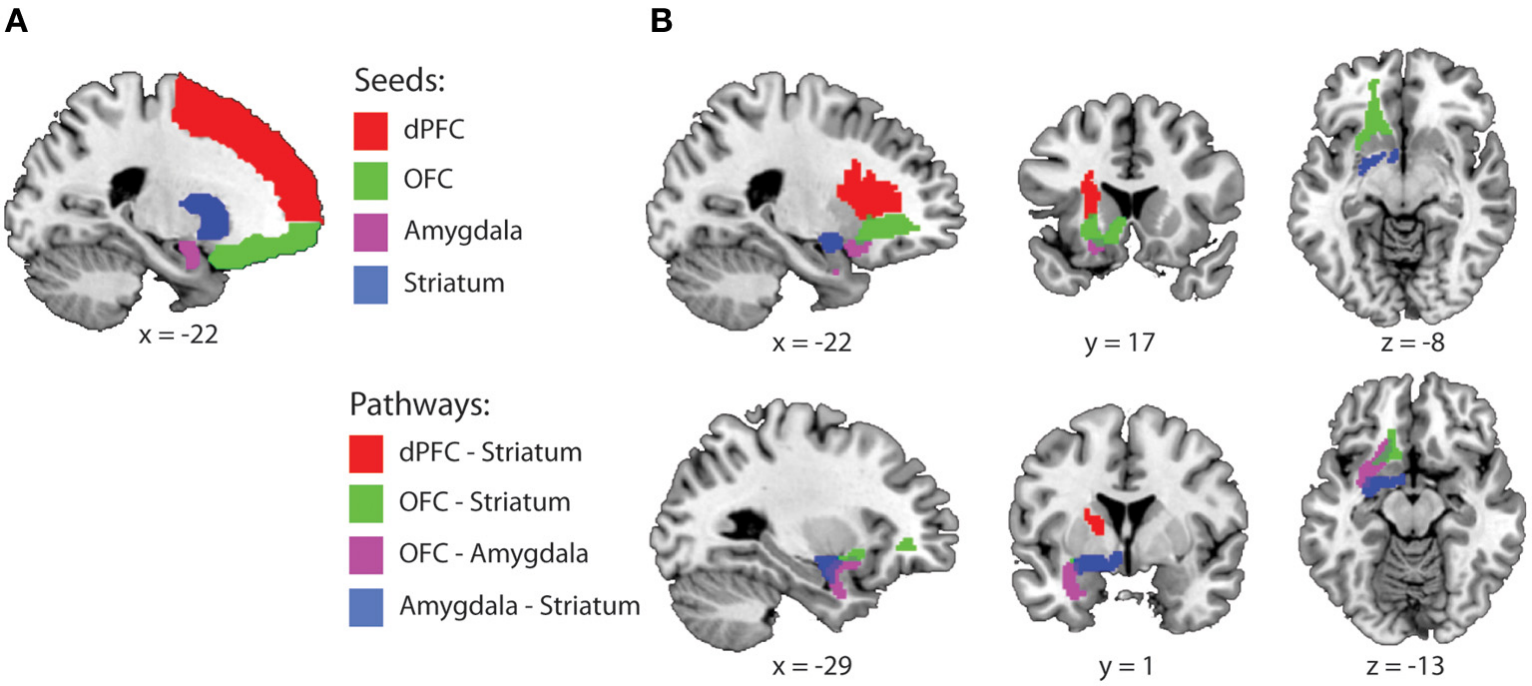
**Fronto-striatal-limbic pathways. (A)** Seed and waypoint masks that were used for probabilistic tractography, displayed on a MNI-template. **(B)** The four study-specific anatomical ROIs used for FA and MD data extraction. Colored masks represent the binarized group masks for pathways that were present in at least 75% of all subjects. Abbreviations: dPFC, dorsal prefrontal cortex; OFC, Orbitofrontal cortex.

Orbitofronto-limbic-striatal pathways are not yet included in white matter atlases. Accordingly, these study-specific anatomical pathways were created using probabilistic tractography as implemented in FMRIB's diffusion toolbox [See also (De Zeeuw et al., 2012; Mandl et al., 2012; Peper et al., 2013) for similar procedures]. In total four pathways were created; OFC—striatum, OFC—amygdala, amygdala—striatum and dorsal PFC—striatum. Masks used for tractography were defined in standard space using the AAL-template (Tzourio-Mazoyer et al., 2002) (Figure 1A). Because the locus of the drug effects was centered on the left striatum we focussed our analysis on pathways in the left hemisphere. The left amygdala was defined as AAL-region 41, the left OFC as the gyrus rectus and orbito gyrus regions (Ogar and Gorno-Tempini, 2007) (AAL-regions 5, 9, 15, 25 and 2), the left dPFC as all left superior, middle and inferior frontal gyrus regions (AAL-regions 3, 7, 13, 23) and the left striatum as the left putamen and caudate nucleus (AAL-regions 71 and 73). Ventral and dorsal striatal subregions are not clearly separated by anatomical boundaries and best defined by its afferent projections from cortical areas (Haber and Knutson, 2010). Accordingly, we seeded our tractography from the OFC, dPFC and amygdala and used the whole striatum as waypoint mask.

For each pathway, waypoint (a.k.a. inclusion) and exclusion masks were defined as followed: (1) *OFC-Striatum*: Seed = OFC, waypoint = striatum, exclusion = dPFC, amygdala and planes excluding *x* > 1 and *y* < −18. (2) *Amygdala-Striatum*: Seed = amygdala, waypoint = striatum, exclusion = OFC, dPFC and planes excluding *x* > 1 and y < −18. (3) *OFC-Amygdala*: Seed = OFC, waypoint = amygdala, exclusion = dPFC, striatum, and planes excluding *x* > 1, and *y* < −18. (4) *dPFC-striatum*: Seed dPFC, waypoint: striatum, exclusion: OFC, amygdala and planes excluding *x* > 1 and *y* < −18. These masks were brought back into native space, using the inverse of the computed normalization parameters to create individual probabilistic diffusion pathways. Using FMRIB's diffusion toolbox [FMRIB's Software Library (FSL), bedpostx], fiber orientation probabilistic density functions were estimated at each voxel, allowing for multiple fiber directions (Behrens et al., 2007). Five thousand streamline samples per seed voxel were drawn through the probability density functions to form an estimate of the probability distribution of connections from each seeded voxel (“probtrackx” with a curvature threshold of 0.2). All pathways from the seed region that passed through the exclusion mask and all pathways that did not pass through the waypoint mask were discarded from the calculation of the connectivity distribution. The resulting connectivity distribution files are images in which the values at each voxel represent the number of samples between the seed and waypoint mask that passed through that voxel. These images were then brought back to standard space, using individual normalization parameters, thresholded to include voxels through which at least 1% of the samples passed, binarized and summed across subjects. The 4 study-specific anatomical VOI's were created at the group level representing those pathways that were present in at least 75% of all subjects (Figure 1B). These are commonly used thresholds and are similar or more conservative compared with thresholds used in other fibre-based DTI tractography studies (Leh et al., 2007; Gutman et al., 2009; Mandl et al., 2012; Peper et al., 2013). Finally, the individual mean FA and MD values were extracted from each pathway.

### Statistical analysis

Because we used different scanning protocols, the extracted FA and MD values of each pathway were first residualized with respect to protocol. Multiple linear regression analysis (SPSS, version 19.0.0) was done with the residualized FA values from the four pathways as predictor variables and the drug-related difference in beta values (BOLD) as dependent variable. A stepwise procedure was applied to include only those predictors that significantly contributed the model. The probability to enter or remove a predictor was set at 0.05 and 0.1, respectively (default). Consistent with our previous report, assessments of the different drug comparisons were done in a fixed a priori defined order. First, we investigated which of the pathways contributed to the effects of dopamine receptor stimulation (bromocriptine) relative to dopamine receptor blockade (sulpiride) on striatal BOLD. Next, for the pathways that were revealed in the first step, we assessed whether their contribution was driven by effects of bromocriptine relative to placebo or by effects of sulpiride relative to placebo. Finally, to establish the D2 receptor dependency of the observed effects, we assessed whether they were blocked by combined administration. The same procedures were used to assess associations between drug effects on behavior and FA values from these pathways. To further support the nature of our FA findings we also assessed the association between drug effect on BOLD and MD values in the pathways that yielded a significant relationship from the analysis described above. While FA values represent the orientation-dependence of water diffusion, which is directional in white matter fibers, MD values represent the overall magnitude of water diffusion. MD depends on fiber and membrane density and, in white matter, increases in MD have been associated with the degeneration of fiber bundles (Beaulieu, 2002; Thomason and Thompson, 2011). Accordingly, when, across subjects, higher FA values in white matter are accompanied by lower MD values, this likely reflects higher levels of fiber and membrane density within non-crossing fiber bundles. Conversely, when across subjects, higher FA values are accompanied by higher MD values, this possibly reflects selectively lower levels of fiber and membrane density within of one of the fiber bundles in a crossing fiber region.

Finally, for completion, main effects of drugs on behavior were assessed with repeated measures ANOVA with the within-subjects factors drug and valence (reward and punishment). The order of drug comparisons were assessed in the same a priori defined order as described above.

### Supplementary analysis

In addition to the volume of interest analyses, we conducted supplementary voxel-wise regression analysis at the whole-brain level, using random effects multiple regression procedures in SPM8 (http://www.fil.ion.ucl.ac.uk/spm). This allowed us to visualize the (physiological plausibility of) effects that were revealed to be statistically significant using the volume of interest analyses. To this end, individual FA-maps were submitted to a second-level one sample *T*-test and the drug-related changes in striatal BOLD signal were entered as a covariate of interest. Scan-protocol was entered as a covariate of no-interest. Voxels revealed by the covariate of interest represent white matter regions that exhibit a linear relationship between individual FA-values and drug effects on striatal BOLD. Effects are displayed for visualization purposes only at a threshold of *p* < 0.001 uncorrected for multiple comparisons (Figure 3). Next, probabilistic diffusion tractography (FMRIB's diffusion toolbox) was used to visualize the pathways connecting with the FA region revealed by the voxel-wise regression analysis as a seed. To this end, the FA seed region was defined as a 4 mm sphere around the peak voxel of the FA region (*x, y, z* = −34, 8, −6) revealed by the regression analysis. For each subject, this region was brought back into native space, using the inverse of the computed normalization parameters and used as a seed region for probabilistic tractography (same settings as above). The resulting connectivity distribution images were brought back to standard space, using individual normalization parameters, and tractography maps were thresholded to include only voxels through which at least 1% of all samples had passed. These individual maps were then binarized and summed across subjects to produce group probability maps.

## Results

**Figure 2:**
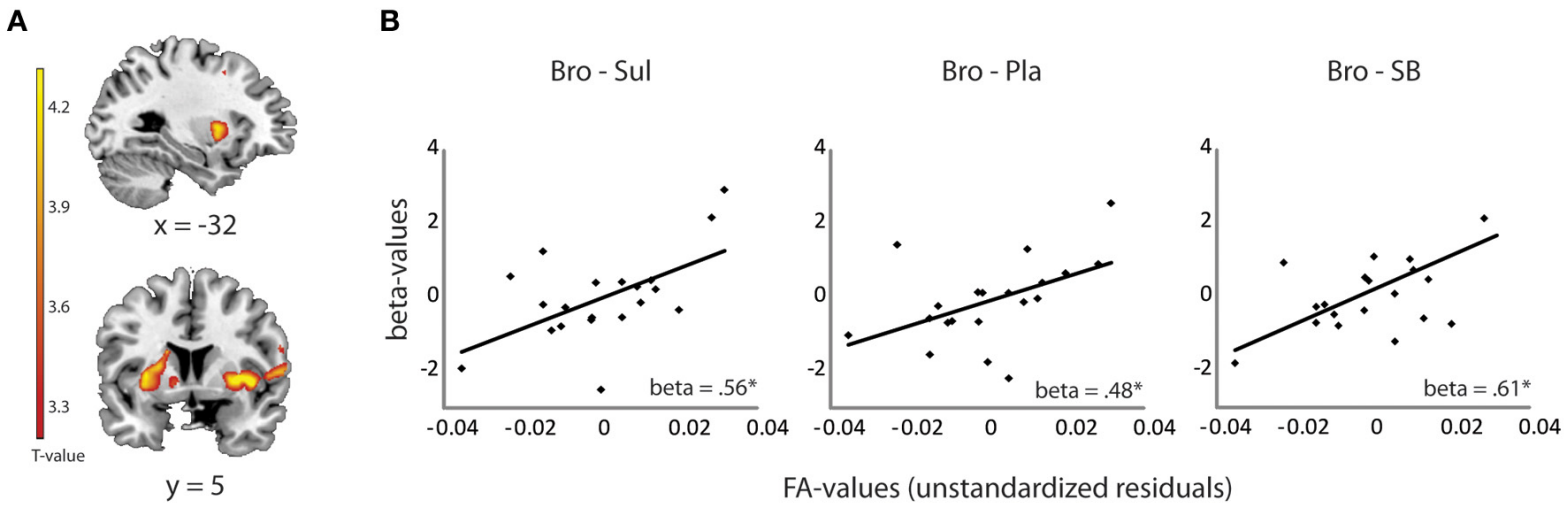
**Relationship between drug effects on BOLD and FA-values, revealed by ROI analyses. (A)** Shown are effects of bromocriptine relative to sulpiride on striatal BOLD signal during unexpected outcomes in the reversal learning task [(unexpected—expected rewards) + (unexpected—expected punishments)] (*x, y, z* = −22, 18, 4, *p*_fwe_striatum_ = 0.03). These effects were reported previously in Van Der Schaaf et al. (2012) and depended on working memory capacity. **(B)** Linear relationship between FA values in the OFC-amygdala pathway and the effects of bromocriptine relative to sulpiride (left), bromocriptine relative to placebo (middle) and bromocriptine relative to combined administration of both drugs (right) on striatal BOLD. Only significant effects are shown. ^*^p < 0.05 Abbreviations: Pla, placebo; Bro, bromocriptine; Sul, sulpiride; SB = combined administration of bromocriptine and sulpiride.

Linear regression analysis revealed a significant positive relationship between drug effects on striatal BOLD (bromocriptine—sulpiride) and FA values from the OFC-amygdala pathway [*F*_(1, 19)_ = 8.33, *R*^2^ = 0.32, adjusted *R*^2^ = 0.28, β = 0.56, *T* = 2.89, *p* = 0.010]. No significant contribution of the OFC-striatum (β = 0.06, *T* = 0.22, *p* = 0.82), dPFC-striatum (β = 0.01, *T* = −0.03, *p* = 0.98) or the amygdala-striatum pathways (β = −0.04, *T* = −0.18, *p* = 0.86) were revealed. FA values from the OFC-amygdala pathway were associated with the effects of bromocriptine relative to placebo on striatal BOLD [*F*_(1, 19)_ = 5.63, *R*^2^ = 0.24, adjusted *R*^2^ = 0.19, β = 0.48, *T* = 2.37, *p* = 0.029], but not of sulpiride relative to placebo (beta = −0.14, *p* = 0.55). Moreover, these white-matter dependent effects of bromocriptine on striatal BOLD were abolished by co-administration of both drugs; FA-values from the OFC—amygdala pathway correlated significantly with the effects of bromocriptine relative to combined administration on striatal BOLD [*F*_(1, 19)_ = 10.73, *R*^2^ = 0.37, adjusted *R*^2^ = 0.34, β = 0.61, *T* = 3.28, *p* = 0.004], but not with the effects of placebo relative to combined administration on striatal BOLD (β = −0.23, *p* = 0.34) (Figure 2B).

Subsequent correlation analyses with MD-values from the OFC–amygdala pathway revealed a negative relationship between the effect of bromocriptine relative to sulpiride on striatal BOLD and MD-values from the OFC-amygdala pathway (β = −0.61, *p* = 0.004). Thus, the found associations with FA were accompanied by associations with fiber density within the OFC-amygdala pathway. Taken together, these data show that bromocriptine increased reversal-related striatal BOLD in subjects with high FA-values in the OFC-amygdala pathway, while it decreased striatal BOLD in subjects with low FA-values in this pathway. These effects were likely mediated via D2 receptor stimulation, as effects were abolished by co-administration with sulpiride.

There were no associations between FA-values and drug effects (reported here are effects of bromocriptine relative to placebo) on behavioral measures of valence-dependent reversal learning (OFC-amygdala: β = −0.10, *p* = 0.68; OFC-striatum: β = −0.23, *p* = 0.34; dPFC-striatum: β = −0.03, *p* = 0.89; Amygdala-striatum: β = −0.27, *p* = 0.25) or valence-independent reversal learning (OFC-amygdala: β = −0.34, *p* = 0.14; OFC-striatum: β = −0.23, *p* = 0.33; dPFC-striatum: β = −0.25, *p* = 0.30; amygdala-striatum: β = −0.06, *p* = 0.81).

For completeness, we also assessed drug effects on behavior irrespective of FA values. This revealed a trend toward opposite effects of bromocriptine and sulpiride on reward and punishment reversal learning [drug × valence: *F*_(1, 19)_ = 4.2, *P* = 0.054]. This was due to better punishment relative to reward learning after bromocriptine (raw accuracy scores ± standard error of the mean: reward: 0.90 ± 0.02; punishment: 0.92 ± 0.01), but better reward relative to punishment learning after sulpiride (reward: 0.93 ± 0.02; punishment: 0.90 ± 0.02). However, no drug by valence effects were seen when comparing bromocriptine with placebo (reward: 0.90 ± 0.02; punishment: 0.89 ± 0.03) [drug × valence: *F*_(1, 19)_ = 1.8, *P* = 0.20].

### Supplementary analyses

**Figure 3:**
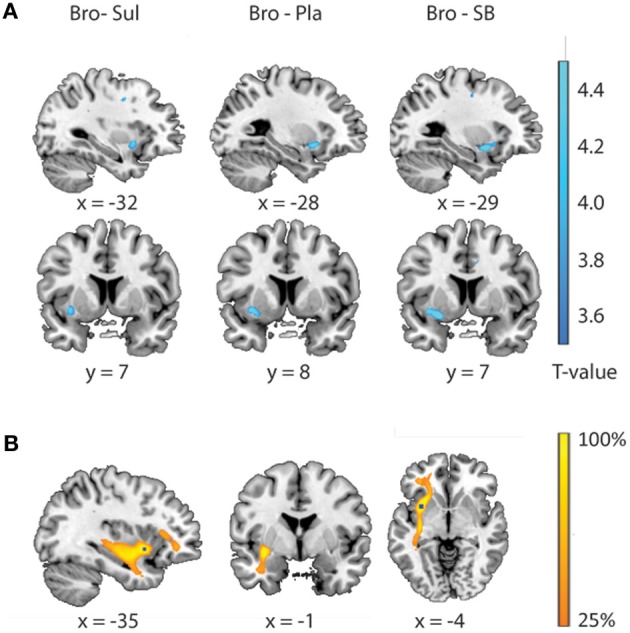
**Relationship between drug effects on BOLD and FA-values, revealed by whole brain analyses. (A)** White matter regions showing a linear relationship between FA-values and the effects of bromocriptine relative to sulpiride (left), bromocriptine relative to placebo (middle) and bromocriptine relative to combined administration of both drugs (right) on striatal BOLD. **(B)** Summed tractography maps of the individual pathways that originate from the FA-region displayed in Figure 3A (left). Probabilistic tractography from this region revealed an extensive network of pathways between the OFC, amygdala and striatum. Image is thresholded to present those tracks that were present in at least 25% of the subjects.

Results from the brain-wide voxel wise regression analyses and subsequent tractography concurred with the results from the volumes of interest analyses reported above. Thus, brain-wide analysis revealed that FA in a region within the uncinate fasciculus, as identified with the JHU white matter tractography atlas, predicted drug effects on striatal BOLD signal (bromocriptine—sulpiride: *x, y, z* = −34, 8, −6, *T* = 4.87, *p*_unc_ < 0.001; bromocriptine—placebo: *x, y, z* = −30, 10, −8, *T* = 3.84, *p*_unc_ < 0.001; bromocriptine-combined: *x, y, z* = −26, 4, −12, *T* = 6.51, *p*_unc_ < 0.001) (Table 1, Figure 3A). Probabilistic tractography from this region revealed an extended network of pathways between the OFC, amygdala and striatum. Other pathways revealed by tractography included connections with the insular cortex and a pathway along the inferior longitudinal fasciculus along the hippocampus and toward the visual cortex. No pathways toward the thalamus or midbrain regions were seen. These tractography findings further support that our findings likely involve white matter integrity in the OFC-amygdala pathway, rather than direct fronto-striatal pathways, as the latter typically also involve thalamic connections (Haber and Knutson, 2010) (Figure 3B).

**Table 1:** **Whole brain results from the voxel-wise regression analysis of FA regions that showed a linear correlation with drug effects on striatal BOLD**.

**Side**	***N* voxels**	***T*-value**	**MNI**		
			***x***	***y***	***z***
**BROMOCRIPTINE—SULPIRIDE (POSITIVE)**					
L	14	5.12	−34	0	42
L	**35**	**4.87**	**−34**	**8**	**−6**
R	16	4.77	26	40	14
L	13	4.43	−10	38	46
L	17	4.34	−30	−20	−46
**BROMOCRIPTINE—PLACEBO (POSITIVE)**					
R	13	5.32	16	−28	44
L	**73**	**3.84**	**−30**	**10**	**−8**
**BROMOCRIPTINE—COMBINED(POSITIVE)**					
L	**196**	**6.51**	**−26**	**4**	**−12**
L	14	4.48	−30	−14	−46
L	11	3.93	−18	−54	12

The regions that fell within our anatomically defined pathways are printed in bold. Data is presented with p < 0.001 uncorrected and extended threshold of >10 voxels.

## Discussion

Dopaminergic drug effects have been shown to vary greatly between individuals (Cools and D'esposito, 2011). Here we provide evidence for an important link between dopaminergic drug effects during reversal learning and neuroanatomical integrity of connections between the OFC and amygdala. More specifically, we demonstrate that dopaminergic drug effects on striatal BOLD signal during reversal learning vary as a function of FA and MD in a pathway connecting the OFC with the amygdala. FA and MD rely on several microstructural properties, including the level of axon myelination, intact axonal membranes and fiber density (Beaulieu, 2002). Accordingly, our results support the hypothesis that dopaminergic drug effects on human striatal BOLD signal are associated with the neuronal communication efficiency of cortico-limbic projections. The implication of these findings is 2-fold. First, the data provide fundamental insight into the mechanism of action of dopaminergic drug effects on reward-related processing and reversal learning. Specifically, effects of D2 receptor stimulation during reversal learning involve an orbitofronto-limbic-striatal network. Second, they may have important clinical implications by suggesting that measures of white matter integrity can help predict dopaminergic drug effects on brain function, thus contributing ultimately to the individual tailoring of dopaminergic drug treatment strategies in psychiatry.

The drug effects on striatal BOLD signal were associated with white matter integrity of the pathway connecting the OFC with the amygdala (i.e., part of the uncinate fasciculus), and not by that of direct orbitofronto-striatal or amygdala-striatal projections. These findings extend previous non-pharmacological human DTI studies demonstrating that reward-related striatal BOLD responses (Camara et al., 2010) and associated functional connectivity (Cohen et al., 2008) are associated with white matter integrity of orbitofrontal-limbic-striatal pathways. Furthermore, we also showed that the drug effects during reversal learning were not associated with white matter integrity of dorsal fronto-striatal connections, which are suggested to be involved in more cognitive and motor processing (Alexander et al., 1990; Haber and Knutson, 2010). Indeed, our findings complement those from a recent study (Van Schouwenburg et al., 2013), in which we demonstrated that white matter integrity of a dorsal fronto-striatal-thalamic pathway was associated with drug effects on striatal BOLD signals during a form of attention-shifting that did not involve reward. Together, these data establish that associations between dopaminergic drug effects and white matter integrity are neuroanatomically specific and depend on task demands.

Our results are consistent with animal lesion work that has repeatedly demonstrated the crucial role of OFC-amygdala interactions in reversal learning (Baxter et al., 2000; Stalnaker et al., 2007; Schoenbaum et al., 2009). The OFC has originally been suggested to rapidly encode new associations and regulate reversal learning by directly driving areas such as the striatum (Thorpe et al., 1983). However, accumulating evidence indicates that the OFC instead contributes indirectly to the updating of stimulus-outcome associations by providing information about expected outcomes to other down-stream areas such as the amygdala (Stalnaker et al., 2007; Schoenbaum et al., 2009; Takahashi et al., 2009). Amygdala projections to the ventral striatum might then, in turn, mediate the effects of (updated) outcome-predictive stimuli on action selection. Indeed, electrophysiological responses in the ventral striatum (and associated behavioral responding) to relevant sensory stimuli critically depend on concomitant amygdala and dopamine inputs (Robbins et al., 1989; Everitt et al., 1991; Ambroggi et al., 2008; Pennartz et al., 2011). Accordingly, our results highlight the importance of indirect OFC-amygdala pathways in reversal learning by showing that dopaminergic modulation of striatal BOLD responses during reversal learning are not associated with white matter integrity of direct fronto-striatal pathways, but instead are associated with white matter integrity of the OFC-amygdala pathway. Together, these results provide fundamental insight into the mechanism by which dopamine changes brain function during reversal learning.

In addition, our experimental design allowed us to establish that these white-matter dependent drug effects were mediated by D2 receptors. Effects of the D2 receptor agonist bromocriptine on striatal BOLD signal were abolished by co-administration with the D2 receptor antagonist sulpiride. This generally concurs with animal work demonstrating that reversal learning in monkeys is selectively mediated by D2 receptors but not D1 receptors (Lee et al., 2007). In addition, animal work has demonstrated that the effects of dopamine on the output of amygdala neurons are at least partially mediated by D2 receptors (Rosenkranz and Grace, 1999, 2002a; Grace and Rosenkranz, 2002; Bissière et al., 2003). While D2 receptor stimulation was found to potentiate sensory driven amygdala outputs to the striatum, D1 receptor stimulation was found to attenuate PFC inhibitory influences on amygdala output neurons (Rosenkranz and Grace, 1999, 2002a). Based on such experimental animal work, we speculate that bromocriptine potentiated sensory-driven amygdala output excitability to a greater extent in subjects with high communicational efficacy within the OFC-amygdala pathway than in those with low OFC-amygdala connectivity. It might be noted we cannot provide definitive evidence for this latter hypothesis, because DTI is inconclusive with regard to the direction in which information travels. Nevertheless, our results do converge with prior animal work and highlight the importance of D2 receptor stimulation for reversal learning.

One caveat of our study is that we did not find evidence for a direct relationship between white matter integrity of the OFC-amygdala pathway and drug effects on behavioral updating of stimulus-outcome associations. This is particularly surprising given that experimental animal work has demonstrated that the OFC and amygdala (Iversen and Mishkin, 1970; Jones and Mishkin, 1972) and their interaction (Baxter et al., 2000; Stalnaker et al., 2007) are crucial for behavioral performance on reversal learning tasks. Accordingly, we believe that our failure to observe correlations with drug effects on behavior might reflect a relative lack of sensitivity. Future work should reveal whether the present finding that white matter integrity of orbitofrontal-limbic-striatal pathways is associated with drug effects on brain function extends to behavior.

### Conflict of interest statement

The authors declare that the research was conducted in the absence of any commercial or financial relationships that could be construed as a potential conflict of interest.
